# Plant-based insect repellents: a review of their efficacy, development and testing

**DOI:** 10.1186/1475-2875-10-S1-S11

**Published:** 2011-03-15

**Authors:** Marta Ferreira Maia, Sarah J Moore

**Affiliations:** 1 Disease Control Department, London School of Hygiene and Tropical Medicine, Keppel Street, London WC1E 7HT, UK; 2 Biomedical and Environmental Thematic Group, Ifakara Health Institute, Ifakara, Morogoro, Tanzania

## Abstract

Plant-based repellents have been used for generations in traditional practice as a personal protection measure against host-seeking mosquitoes. Knowledge on traditional repellent plants obtained through ethnobotanical studies is a valuable resource for the development of new natural products. Recently, commercial repellent products containing plant-based ingredients have gained increasing popularity among consumers, as these are commonly perceived as “safe” in comparison to long-established synthetic repellents although this is sometimes a misconception. To date insufficient studies have followed standard WHO Pesticide Evaluation Scheme guidelines for repellent testing. There is a need for further standardized studies in order to better evaluate repellent compounds and develop new products that offer high repellency as well as good consumer safety. This paper presents a summary of recent information on testing, efficacy and safety of plant-based repellents as well as promising new developments in the field.

## Background

Most plants contain compounds that they use in preventing attack from phytophagous (plant eating) insects. These chemicals fall into several categories, including repellents, feeding deterrents, toxins, and growth regulators. Most can be grouped into five major chemical categories: (1) nitrogen compounds (primarily alkaloids), (2) terpenoids, (3) phenolics, (4) proteinase inhibitors, and (5) growth regulators. Although the primary functions of these compounds is defence against phytophagous insects, many are also effective against mosquitoes and other biting Diptera, especially those volatile components released as a consequence of herbivory [1]. The fact that several of these compounds are repellent to haematophagous insects could be an evolutionary relict from a plant-feeding ancestor, as many of these compounds evolved as repellents to phytophagous insects [2], and this repellent response to potentially toxic compounds is well conserved in the lineage of Diptera (True Flies). Insects detect odours when that volatile odour binds to odorant receptor (OR) proteins displayed on ciliated dendrites of specialized odour receptor neurons (ORNs) that are exposed to the external environment, often on the antennae and maxillary palps of the insect, and some ORNs, such as OR83b that is important in olfaction and blocked by the gold-standard synthetic repellent DEET (N, N-diethyl-3-methylbenzamide) [3], are highly conserved across insect species [4,5]. Plants commonly produce volatile “green leaf volatiles” when leaves are damaged in order to deter herbivores [6], and several authors have shown strong responses of mosquito odour receptors to this class of volatiles including geranyl acetate and citronellal [7], 6-methyl-5- hepten-2-one and geranylacetone [8]. Interestingly, the same odour receptors that respond to DEET also respond to thujone eucalyptol and linalool in *Culex quinquefasciatus*[9]. In *Anopheles gambiae*, the DEET receptor OR83b is stimulated by citronellal, but is also modulated by the TRPA1 cation channel [10]. However, it is most likely that many plant volatiles are deterrent or repellent because they have high vapour toxicity to insects [11,12].

**Figure 1:**
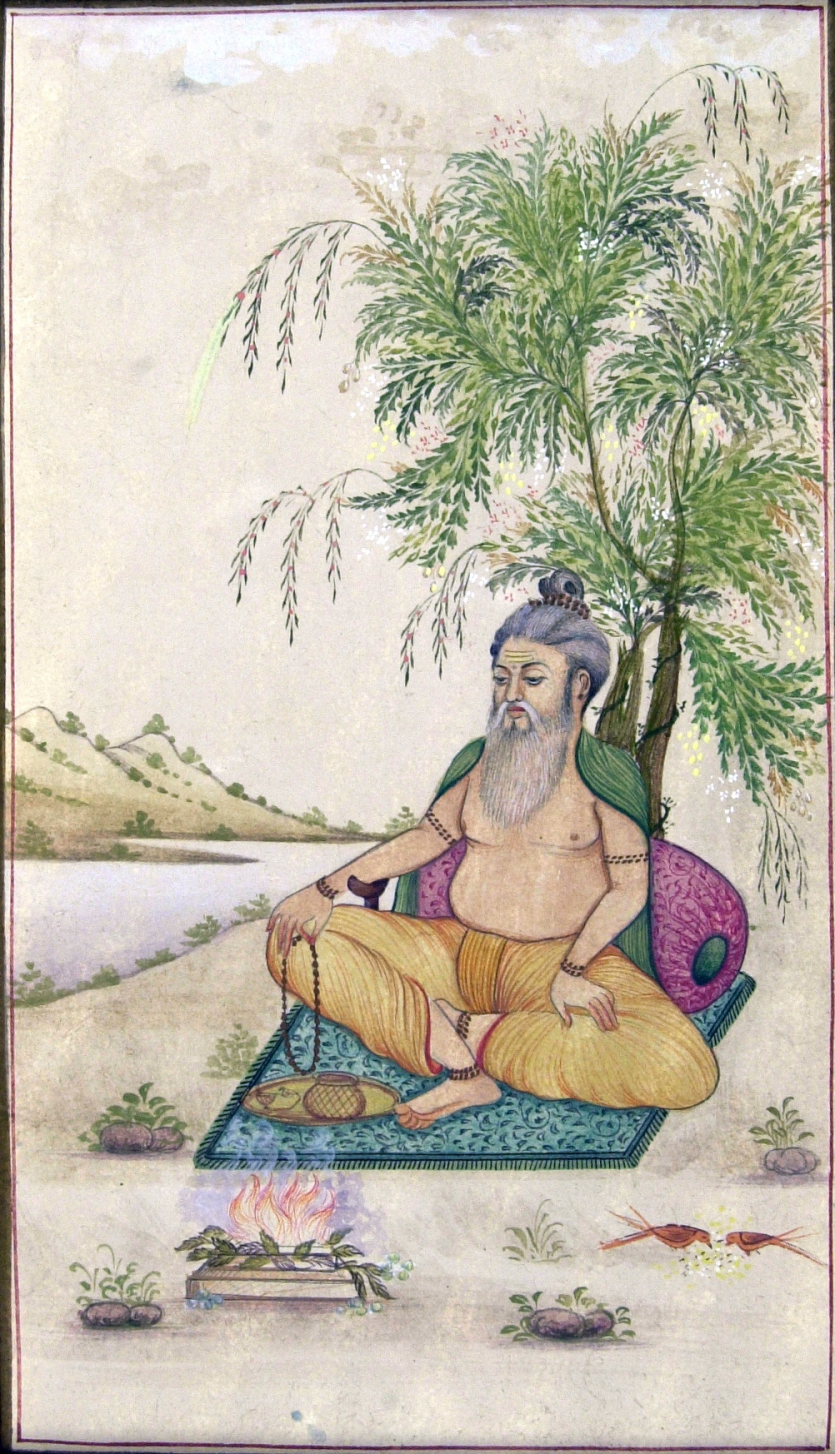
Moghul painting illustrating a man burning neem leaves near a river where biting insects would be present (^©^ Dr Sarah Moore)

This repellency of plant material has been exploited for thousands of years by man, most simply by hanging bruised plants in houses, a practice that is still in wide use throughout the developing countries [13]. Plants have also been used for centuries in the form of crude fumigants where plants were burnt to drive away nuisance mosquitoes and later as oil formulations applied to the skin or clothes which was first recorded in writings by ancient Greek [14], Roman [15] and Indian scholars [16] (Figure 1). Plant-based repellents are still extensively used in this traditional way throughout rural communities in the tropics because for many of the poorest communities the only means of protection from mosquito bites that are available [13], and indeed for some of these communities [17], as in the Europe and North America [18] “natural” smelling repellents are preferred because plants are perceived as a safe and trusted means of mosquito bite prevention.

**Figure 2:**
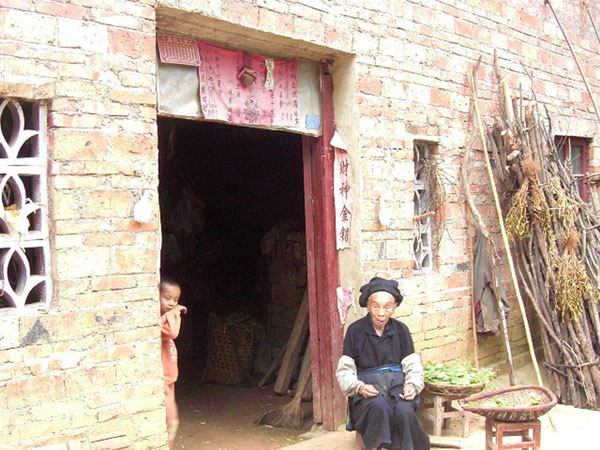
A village herbalist in rural Yunnan, Southern China. This lady was a key informant for an ethnobotanical study into plants used to repel mosquitoes (^©^ Dr Sarah Moore)

The discovery of new plant-based repellents is heavily reliant on ethnobotany. This is the targeted search for medicinal plants through in-depth interviews with key informants knowledgeable in folk-lore and traditional medicine. It is common practice to conduct ethnobotanical surveys using structured interviews, combined with the collection of plant voucher Specimens (Figure 2), to evaluate plant use by indigenous ethnic groups [19]. Questions are asked about plant usage, abundance and source. This is a more direct method of identifying plants with a potential use than general screening of all plants in an area. A second means is bio-prospecting, where plants are systematically screened for a particular mode of action, which is a costly and labour intensive means of identifying new repellents. However, mass screening of plants for repellent activity was the way by which PMD (para-methane 3-8, diol), an effective and commercially available repellent was discovered in the 1960s [20].

## PMD from lemon eucalyptus (*Corymbia citriodora*) extract

*Corymbia citriodora* (Myrtaceae), also known as lemon eucalyptus, is a potent natural repellent extracted from the leaves of lemon eucalyptus trees (Table 1). It was discovered in the 1960s during mass screenings of plants used in Chinese traditional medicine. Lemon eucalyptus essential oil, comprising 85% citronellal, is used by cosmetic industries due to its fresh smell [21]. However, it was discovered that the waste distillate remaining after hydro-distillation of the essential oil was far more effective at repelling mosquitoes than the essential oil itself. Many plant extracts and oils repel mosquitoes, with their effect lasting from several minutes to several hours (Table 1). Their active ingredients tend to be highly volatile, so although they are effective repellents for a short period after application, they rapidly evaporate leaving the user unprotected. The exception to this is para-menthane 3, 8 diol, which has a lower vapour pressure than volatile monoterpines found in most plant oils [22] and provides very high protection from a broad range of insect vectors over several hours [23], whereas the essential oil is repellent for around one hour [24]. PMD is the only plant-based repellent that has been advocated for use in disease endemic areas by the CDC (Centres for Disease Control) [25], due to its proven clinical efficacy to prevent malaria [26] and is considered to pose no risk to human health [27]. It should be noted that the essential oil of lemon eucalyptus does not have EPA (Environmental Protection Agency) registration for use as an insect repellent.

**Table 1:** An overview of repellent plant efficacy from literature review

Plant	Location	Other names	Repellent compound(s)	Tested mode of use	Repellency % protection	Study type	Ref
MYRTACEAE							
*Corymbia citriodora*	Australia Brazil Bolivia China India Ethiopia Tanzania Kenya	lemon eucalyptus lemon scented gum quwenling	citronellal PMD (by product of hidrodistillation) (p-menthane-3,8-diol) citronellol limonene geraniol isopulegol δ-pinene	30% PMD applied topically	96.88% protection from mosquitoes for 4 hours	field study in Bolivia	[35]
				PMD towelette (0.575g) applied topically	90% protection from *An. arabiensis* for 6 hours	laboratory study	[95]
				50% PMD applied topically	100% protection from *An gambiae* and *An. funestus* for 6-7 hours	field study in Tanzania	[96]
				20% PMD (1.7 mg/cm^2^) applied topically	100% protection for 11-12 hours against *A. stephensi*	laboratory study	[52]
				20% PMD applied topically	100% protection against *Ae. Aegypti* for 120 minutes	Laboratory study	[42]
				thermal expulsion (leaves)	78.7 % protection from *An. arabiensis* 76.8% protection from *An. pharaoensis*	field study in Ethiopia	[97]
				direct burning (leaves)	70.1 % protection from *An. arabiensis* 72.9% protection from *An. pharaoensis*	field study in Ethiopia	[97]
				periodic thermal expulsion (leaves)	74.5% protection from *An. gambiae* s.s.	semi-field study in Kenya	[50]
				periodic direct burning (leaves)	51.3% protection from *An. gambiae* s.s.	semi-field study in Kenya	[50]
				thermal expulsion (leaves)	48.71% protection from *An. gambiae s.l.*	field study in Kenya	[98]
*Eucalyptus* spp.	Guinea-Bissau Ethiopia Tanzania Portugal	eucalyptus	1,8-cineole citronellal Z- and α- citral α-pinene	thermal expulsion (leaves)	72.2% protection from mosquitoes for 2 hours	field study in Guinea Bissau	[99]
*E. camaldulensis*	Ethiopia			thermal expulsion (leaves)	71.9 % protection from *An. arabiensis* 72.2% protection from *An. pharaoensis*	field study in Ethiopia	[97]
				direct burning (leaves)	65.3 % protection from *An. arabiensis* 66.6% protection from *An. pharaoensis*	field study in Ethiopia	[97]
*Eugenia caryophyllus* or *Syzygium aromaticum* or *Eugenia aromaticu*	India	clove lavang cravinho-da-india	Eugenol carvacrol thymol cinnamaldehyde	100% essential oil applied topically	100% protection against *Ae. aegypti* for 225 minutes 100% protection against *An. albimanus* for 213 minutes	laboratory study	[53]
				100% essential oil applied topically	100% protection against *Ae. aegypti* for 120 min. 100% protection against *C. quinquefasciatus* for 240 min. 100% protection against *An. dirus* for 210 min.	laboratory study	[23]
VERBENACEAE							
*Lippia* spp.	Kenya Tanzania Ghana Zimbabwe	lemon bush	myrcene linalool α-pinene eucalyptol				
*L. javanica*			alloparinol camphor limonene α –terpeneol verbenone	5mg/cm2 plant extract applied topically	100% protection against *Ae. aegypti for 8 hours*	laboratory study	[100]
				alcohol plant extract applied topically	76.7% protection against *An arabiensis* for 4 hours	laboratory study	[101]
*L. uckambensis*		fever tea		potted plant	33.3% protection against *An. gambiae* s.s	semi-field study in Kenya	[102]
				periodic thermal expulsion (leaves)	45.9% protection against *An. gambiae* s.s.	semi-field system in Kenya	[50]
				periodic direct burning (leaves)	33.4% protection against *An. gambiae* s.s	semi-field system in Kenya	[50]
				potted plant	25.01% protection against *An.gambiae* s.l	field study in Kenya	[98]
*L. cheraliera*			eucalyptol caryophyllene ipsdienone p-cymene				
*Lantana camara*	Kenya Tanzania	lantana spanish flag West Indian lantana Wild sage	caryophylene	potted plant	32.4% protection against *An. gambiae* s.s	semi-field study in Kenya	[102]
				potted plant	27.22% protection against *An. gambiae* s.l.	field study in Kenya	[98]
				flower extract in coconut oil	94.5% protection against *Ae. aegypti* and *Ae. albopictus* for one hour	laboratory study	[103]
				periodic thermal expulsion (leaves)	42.4% protection against *An. gambiae* s.s	semi-field study in Kenya	[50]
LAMIACEAE							
*Ocimum* spp. *O.americanum*	Kenya Tanzania Zimbabwe Nigeria Ghana Cameroon Eritrea Ethiopia (…)	Tree basil nchu avum lime basil kivumbasi Myeni madongo African blue basil hairy basil	p-cymene estragosl linalool linoleic acid eucalyptol eugenol camphor citral thujone limonene ocimene and others	potted plant	39.70% protection against *An. gambiae* s.s	semi-field study in Kenya	[102]
				potted plant	37.91% protection against *An. gambiae* s.l.	field study in Kenya	[98]
				fresh plants combined with *O. suave* bruised and applied topically	50% protection against *An. gambiae* s.l.	field study in Tanzania	[104]
				periodic thermal expulsion (leaves and seeds)	43.1.% protection against *An gambiae* s.s	semi-field study in Kenya	[50]
				periodic direct burning (leaves and seeds)	20.9% protection against *An. gambiae* s.s	semi-field study in Kenya	[50]
				100% essential oil combined with vanillin 5% applied topically	100% protection against *Ae. aegypti* for 6.5 hours1 100% protection against *C. quinquefasciatus* for 8 hours 100% protection against *An. dirus* for 8 hours	laboratory study	[26]
*O. suave*				thermal expulsion (leaves)	73.6 % protection from *An. arabiensis* 75.1% protection from *An. pharaoensis*	field study in Ethiopia	[97]
				direct burning (leaves)	71.5 % protection from *An. arabiensis* 79.7% protection from *An. pharaoensis*	field study in Ethiopia	[97]
				periodic thermal expulsion (leaves and seeds)	53.1% protection from *An. gambiae* s.s.	semi-field study in Kenya	[50]
				periodic direct burning (leaves and seeds)	28.0% protection from *An. gambiae* s.s.	semi-field study in Kenya	[50]
*O. basilicum*				thermal expulsion (leaves)	78.7 % protection from *An. arabiensis* 79.2% protection from *An. pharaoensis*	field study in Ethiopia	[97]
				direct burning (leaves)	73.1 % protection from *An. arabiensis* 70.0% protection from *An. pharaoensis*	field study in Ethiopia	[97]
				100% essential oil applied topically	100% protection for 70 minutes	laboratory study	[23]
*O. kilimandscharikum*				thermal expulsion (leaves and seeds)	44.54% protection against *An. gambiae* s.l.	field study in Kenya	[98]
				thermal expulsion (leaves and seeds)	37.63% protection against *An. funestus*	field study in Kenya	[98]
				periodic thermal expulsion (leaves and seeds)	52.0% protection against An. gambiae s.s.	semi-field study in Kenya	[50]
				periodic direct burning (leaves and seeds)	26.4% protection against *An. gambiae* s.s	semi-field study in Kenya	[50]
*O. forskolei*				fresh plants hung indoors	53% protection against mosquitoes entering human dwelling	field study in Eritrea	[105]
*Hyptis spp. Hyptis suaveolens*	Kenya Tanzania Ghana The Gambiae	bushmint wild hops wild spikenard hangazimu hortelã-do-campo	myrcene	smouldering on charcoal	85.4% repellency against mosquitoes for 2 hours	field study in Guinea Bissau	[99]
				fresh leaves	73.2% repellency against mosquitoes for 2 hours	field study in Guinea Bissau	[99]
				periodic direct burning (leaves and flowers)	20.8% repellency against *An. gambiae* s.s	semi-field study in Kenya	[50]
*Mentha* spp. *M. piperata*	Brazil Bolivia	hortelã-do-campo peppermint		100% essential oil applied topically	100% protection against *Ae. aegypti* for 45 minutes	laboratory study	[53]
*M. arvensis*		menta Japanese mint		100% essential oil volatilized in a kerosene lamp	41% protection indoors against *Mansonia* spp	field study in Bolivia	[9]
*Thymus* spp*. Th. vulgaris*	China Former Soviet Union Korea Middle-East Mediterranean	thyme	α-terpinene carvacrol thymol p-cymene linalool geraniol	α-terpinene topically	97.3% protection against *Culex pipiens sallens* for 82 min	laboratory study	[106]
				carvacrol topically	94.7% protection against *C. pipiens sallens* for 80 min		
				thymol topically	91.8% protection against *C. pipiens sallens* for 70 min	laboratory study	[106]
				linalool topically	91.7% protection agains *C. pipiens sallens* for 65 min		
				p-cymene	89.0% protection agains *C. pipiens sallens* for 45.2 min		
				100% essential oil applied topically	100% protection against *An. albimanus* for 105 minutes and *Ae. aegypti* for 135 minutes	laboratory study	[53]
				direct burning (leaves)	85-09% protection for 60-90 min	field study	[12]
*Pogostemon spp.*	China	Patchouli		100% essential oil applied	100% protection against *Ae. aegypti* for 120 min	laboratory study	[23]
*Pogostemon cablin*	India Malaysia Thailand	Oriza		topically	100% protection against *C. quinquefasciatus* for 150 min 100% protection against *An. dirus* for 710 minutes		
POACEAE							
*Cymbopogon* spp.	China India Indonesia						
*C. nardus*	Brazil		citronellal	40% essential oil applied topically	100% protection for 7-8 hours against *An. stephensi*	laboratory study	[52]
				100% essential oil applied topically	100% protection against *Ae. aegypti* for 120 min 100% protection against *C. quinquefasciatus* for 100 min 100% protection against *An. dirus* for 70 minutes	laboratory study	[23]
				10% applied topically	100% protection against *Ae. aegypti* for 20 minutes	laboratory study	[42]
*C. martini*	Tanzania Kenya	palmarosa	geraniol	topically (100% essential oil)	100% protection against *An. culicifacies* for 12 hours 96.3% protection against *C. quinquefasciatus* for 12 hours	field study in India	[107]
				topically (100% essential oil)	98.8% protection against *C. quinquefasciatus* for 10 hours	laboratory study	[107]
*C. citratus*	USA South África Bolívia	lemongrass oil grass	citral α-pinene	topically	74% protection against *An. darlingi* for 2.5h 95% protection against *Mansonia* spp. for 2.5 hours	field study in Bolivia	[9]
				Methanol leaf extract applied topically (2.5mg/m^2^)	78.8 % protection against *An. arabiensis* for 12 hours	laboratory study	[108]
				100% essential oil applied topically	100% protection for 30 minutes	laboratory study	[23]
*C. winterianius*				100% essential oil combined with vanillin 5% applied topically	100% protection against *Ae. aegypti* for 6.5 hours 100% protection against *C. quinquefasciatus* for 8 hours 100% protection against *An. dirus* for 8 hours	laboratory study	[26]
*C. excavatus*				alcohol plant extract applied topically	66.7% protection against *An. arabiensis* for 3 hours	laboratory study	[101]
*Pelargonium reniforme*		rose geranium		alcohol plant extract applied topically	63.3 protection against *An. arabiensis* for 3 hours	laboratory study	[101]
MELIACEAE							
*Azadirachta indica*	India Sri Lanka China Brazil Bolívia Pakistan Ethiopia Guinea Bissau Kenya Tanzania (…)	Neem	azadirachtin saponins	direct burning (leaves)	76.0% protection from mosquitoes for 2 hours	field study in Guinea Bissau	[99]
				periodic thermal expulsion (leaves)	24.5% protection from *An. gambiae* s.s	semi-field study in Kenya	[50]
				1% neem oil volatilized in a kerosene lamp	94.2% protection from *Anopheles* spp. 80% protection from *Culex* spp.	field study in India	[109]
				2% neem oil applied topically	56.75% protection from mosquitoes for 4 hours	field study in Bolivia	[35]
ASTERACEAE							
*Tagetes minuta*	Uganda Zimbabwe India	Khaki weed		topically	86.4% protection againt *An. stepehensi* for 6 hours	laboratory study	[110]
				topically	84.2% protection against *C. quinquefasciatus* for 6 hours	laboratory study	[110]
				topically	75% protection against *Ae. aegypti* for 6 hours	laboratory study	[110]
				fresh leaves (4Kg)	reduced human landings indoors	field study in Uganda	[111]
*Artemisia* spp. *A. vulgaris*	India Egypt Italy Canada USA	mugwort wormwood St. Johns plant Old uncle henry Sailors tobacco	camphor linalool terpenen-4-ol α-and β-thujone β-pinene				
*A. monosperma*	Siberia Brazil	Felon herb Naughty man	myrcene limonene cineol	5% leave extract applied topically	100 % protection for 4 hours	field study in Egypt	112
CAESALPINIACEAE							
*Daniellia oliveri*	Guinea-Bissau The Gambiae	churai santão santang santango		direct burning (bark)	77.9% protection against mosquitoes for 2 hours	field study in Guinea Bissau	[99]
				direct burning (bark)	77% protection against mosquitoes	field study in The Gambiae	113
FABACEAE *Glycine max*	Worldwide	Soya		2% soya bean oil	100% protection against *Ae. aegypti* for 95 minutes	laboratoty study	[42]
RUTACEAE *Zanthoxylum limonella*	Thailand	makaen		100% essential oil applied topically	100% protection against *Ae. aegypti* for 120 min 100% protection against *C. quinquefasciatus* for 170 min	laboratory study	[23]
				10% essential oil combined with 10% clove oil	100% protection against *An. dirus* for 190 minutes	laboratory study	[52]
*Citrus hystrix*	Indonesia Malaysia Thailand Laos	Kaffir lime Limau purut		100% essential oil combined with vanillin 5% applied topically	100% protection against *An. stephensi* for 8 hours 100% protection against *Ae. aegypti* for 3 hours 100% protection against *C. quinquefasciatus* for 1.5 hours 100% protection against *An. dirus* for 2.5 hours	laboratory study	[26]
ZINGIBERACEAE *Curcuma longa*		Turmeric Curcuma Indian saffron		100% essential oil combined with vanillin 5% applied topically	100% protection against *Ae. aegypti* for 4.5 hours 100% protection against *C. quinquefasciatus* for 8 hours 100% protection against *An. dirus* for 8 hours	laboratory study	[26]

## Citronella

Essential oils and extracts belonging to plants in the citronella genus (Poaceae) are commonly used as ingredients of plant-based mosquito repellents (Table 1), mainly *Cymbopogon nardus* that is sold in Europe and North America in commercial preparations. Citronella has found its way into many commercial preparations through its familiarity, rather than its efficacy. Citronella was originally extracted for use in perfumery, and its name derives from the French citronelle around 1858 [28]. It was used by the Indian Army to repel mosquitoes at the beginning of the 20^th^ century [29] and was then registered for commercial use in the USA in 1948 [30]. Today, citronella is one of the most widely used natural repellents on the market, used at concentrations of 5-10%. This is lower than most other commercial repellents but higher concentrations can cause skin sensitivity. However, there are relatively few studies that have been carried out to determine the efficacy of essential oils from citronella as arthropod repellents. Citronella-based repellents only protect from host-seeking mosquitoes for about two hours although formulation of the repellent is very important [31,32]. Initially, citronella, which contains citronellal, citronellol, geraniol, citral, α pinene, and limonene, is as effective dose for dose as DEET [33], but the oils rapidly evaporate causing loss of efficacy and leaving the user unprotected. However, by mixing the essential oil of *Cymbopogon winterianus* with a large molecule like vanillin (5%) protection time can be considerable prolonged by reducing the release rate of the volatile oil [34]. Recently, the use of nanotechnology has allowed slower release rates of oils to be achieved, thus prolonging protection time [35]. Encapsulated citronella oil nanoemulsion is prepared by high-pressure homogenization of 2.5% surfactant and 100% glycerol, to create stable droplets that increase the retention of the oil and slow down release. The release rate relates well to the protection time so that a decrease in release rate can prolong mosquito protection time [35]. Another means of prolonging the effect of natural repellents is microencapsulation using gelatin-arabic gum microcapsules, which maintained the repellency of citronella up to 30 days on treated fabric stored at room temperature (22°C) [36]. The use of these technologies to enhance the performance of natural repellents may revolutionize the repellent market and make plant oils a more viable option for use in long-lasting repellents. However, for the time-being travellers to disease endemic areas should not be recommended citronella-based repellents [32]. In contrast, for those communities where more efficacious alternatives are not available, or are prohibitively expensive, the use of citronella to prevent mosquito bites may provide important protection from disease vectors [17].

The second way to use volatile plant repellents is to continuously evaporate them. Citronella and geraniol candles are widely sold as outdoor repellents, however field studies against mixed populations of nuisance mosquitoes show reductions in biting around 50%, although they do not provide significant protection against mosquito bites [37-39].

## Neem

Neem is widely advertised as a natural alternative to DEET [40], and it has been tested for repellency against range of arthropods of medical importance, with variable results (Table 1). Several field studies from India have shown very high efficacy of Neem-based preparations [41-43], contrasting with findings of intermediate repellency by other researchers [44,45]. However, these contrasting results may be due to differing methodologies, and the solvents used to carry the repellents. The EPA has not approved Neem for use as a topical insect repellent. It has a low dermal toxicity, but can cause skin irritation, such as dermatitis when used undiluted [46]. Due to the paucity of reliable studies, Neem oil is not recommended as an effective repellent for use by travellers to disease endemic areas [32], although it may confer some protection against nuisance biting mosquitoes.

## Natural oils and emulsions

Several oils have shown repellency against mosquitoes. It is likely that they work in several ways 1) by reducing short range attractive cues i.e. kairomones, water vapour and temperature [47-49]; 2) by reducing the evaporation and absorption of repellent actives due to the presence of long-chained fatty molecules [50]; 3) by containing fatty acids are known to be repellent to mosquitoes at high concentrations [51]. Bite Blocker, a commercial preparation containing glycerin, lecithin, vanillin, oils of coconut, geranium, and 2% soybean oil can achieve similar repellency to DEET, providing 7.2 hours mean protection time against a dengue vector and nuisance biting mosquitoes in one study [44], and protection for 1.5 hours, equivalent to that of low concentration DEET in a second study [52]. It would appear that the soybean oil in Bite Blocker helps only contributes to repellency as it is not repellent when evaluated on its own [53]. Soybean oil is not EPA registered, but it has low dermal toxicity, although no recommended maximum exposure or chronic exposure limits have been established [54]. Other plant-based oils that have shown some repellent efficacy are coconut oil, palm nut oils [55] and andiroba oil [56], although all of these three oils are far less effective than DEET, they may be useful as carriers for other repellent actives as they are cheap and contain unsaturated fatty acids and emulsifiers that improve repellent coverage and slow evaporation of volatile repellent molecules [50,53,57].

## Essential oils

Essential oils distilled from members of the Lamiaceae (mint family that includes most culinary herbs), Poaceae (aromatic grasses) and Pinaceae (pine and cedar family) are commonly used as insect repellents throughout the globe (Table 1). Many members of these families are used in rural communities through burning or hanging them within homes [58-62]. In Europe and North America there is a strong history of use of the oils dating back to Ancient times. Almost all of the plants used as repellents are also used for food flavouring or in the perfume industry, which may explain the association with these oils as safer natural alternatives to DEET despite many oils causing contact dermatitis (Table 2[63]). Many commercial repellents contain a number of plant essential oils either for fragrance or as repellents including peppermint, lemongrass, geraniol, pine oil, pennyroyal, cedar oil, thyme oil and patchouli. The most effective of these include thyme oil, geraniol, peppermint oil, cedar oil, patchouli and clove that have been found to repel malaria, filarial and yellow fever vectors for a period of 60-180 mins [64-66]. Most of these essential oils are highly volatile and this contributes to their poor longevity as mosquito repellents. However, this problem can be addressed by using fixatives or careful formulation to improve their longevity. For example, oils from turmeric and hairy basil with addition of 5% vanillin repelled 3 species of mosquitoes under cage conditions for a period of 6-8 hours depending on the mosquito species [34]. Although essential oils are exempt from registration through the EPA, they can be irritating to the skin and their repellent effect is variable, dependent on formulation and concentration. Repellents containing only essential oils in the absence of an active ingredient such as DEET should not be recommended as repellents for use in disease endemic areas, and those containing high levels of essential oils could cause skin irritation, especially in the presence of sunlight.

**Table 2:** Some common ingredients in natural repellents that may be hazardous. Reproduced with permission from [63]

Common Name	Scientific Name	Safe Concentration	Hazard
Anise	*Pimpinella anisum*	3.6%	Based on 0.11% methyl eugenol; carcinogen
Basil	*Ocimum* sp	0.07%	Based on 6% methyl eugenol; carcinogen
Bergamot	*Citrus aurantium bergamia*	0.4%	Sensitising and phototoxic; skin irritant
Cajeput	*Melaleuca alternifolia*	0.004%	Based on 97% methyl eugenol; carcinogen
Cedar	*Chamaecyparis nootkatensis*	1%	Likely allergenic contaminants if nootkatone not 98% pure
Cassia	*Cinnamonium cassia*	0.2% or 9%	Sensitising skin irritant
Citronella	*Cymbopogon nardus*	2%	Safety is controversial; based on 0.2% methyl eugenol or 1.3% citral; sensitising skin irritant
Citronella (Java)	*Cymbopogon winterianius*	2%	Based on 0.2% methyl eugenol; carcinogen
Citrus oils	*Citrus* sp	16-25%	Based on 0.005%-0.0025% bergapten; phototoxic skin irritant
Clove	*Syzyguim aromaticum*	0.5%	Based on 92% eugenol; sensitising skin irritant
Fever tea, lemon bush	*Lippia javanica*	2%	Based on 5% citral in related species; sensitising skin irritant
Geranium	*Pelargonium graveolens*	6%	Based on 1.5% citral; sensitising skin irritant
Ginger	*Zingiber* sp	12%	Based on 0.8% citral; sensitising skin irritant
Huon oil, Macquarie pine	*Langarostrobus franklini*	0.004%	Based on 98% methyl eugenol; carcinogen
Lemongrass	*Cymbopogon citratus*	0.1%	Based on 90% citral; sensitising skin irritant
Lime	*Citrus aurantifolia*	0.7%	Phototoxic skin irritant
Litsea	*Litsea cubeba*	0.1%	Based on 78% citral; sensitising skin irritant
Marigold	*Tagates minuta*	0.01%	Phototoxic skin irritant
Mexican tea, American wormseed	*Chenopodium ambrosioides*	Prohibited	Toxic
Mint	*Mentha piperata and spicata*	2%	Based on 0.1% trans-2-hexenal; sensitising skin irritant
Nutmeg	*Myristica fragrans*	0.4%	Based on 1% methyl eugenol; carcinogen
Palmarosa	*Cymbopogon martini*	16%	Based on 1.2% farnesol; sensitizing skin irritant
Pennyroyal	*Mentha pulegium* or *Hedeoma pulegioides*	Prohibited	Toxic
Pine	*Pinus sylvestris*	Prepare with antioxidants	Oxidation creates phototoxic skin irritants
Rosemary	*Rosemarinus officinalis*	36%	Based on 0.011% methyl eugenol; carcinogen
Rue	*Ruta chalepensis*	0.15%	Based on presence of psoralenes; phototoxic skin irritant
Thyme	*Thymus vulgaris*	2%	Based on 0.1% trans-2-hexenal; sensitising skin irritant
Violet	*Viola odorata*	2%	Based on 0.1% trans-2-hexenal; sensitising skin irritant
Ylang-ylang	*Canagium odoratum*	2%	Based on 4% farnesol; sensitizing skin irritant

## Considerations for repellent testing methodology

In a Pubmed search using the terms “plant” and “repellent” and “mosquito” in the past 5 years, 87 results were shown. These studies can be broken down into a series of categories: 1) standard ethnobotanical studies and evaluations of plants that are traditionally used to repel mosquitoes [17,67-70]; 2) standard dose response [33] laboratory evaluations of solvent extractions of plants *without* DEET positive controls [71]; 3) standard dose response [33] laboratory evaluations of solvent or extractions or essential oils of plants *with* DEET positive controls [72] coupled with GC-MS (coupled gas chromatography-mass spectrometry) [73]; 4) laboratory evaluations using time to first bite method [74] comparing the plant repellents to DEET [75] and in addition several of those studies also analysed the constituents of the oil through GC-MS [76,77]. In addition there were a large number of studies that did not use the accepted standard methodology [78] (Table 3), and should be interpreted with caution. Only two studies considered safety [79] or adverse effects [80] and only one study considered randomization and blinding [52], and almost all repellent studies did not consider the number of human participants needed to minimize sampling error [81]. It is important for the future development of plant based repellents that the standard WHO methodology is followed [78], including a DEET control to allow simple comparison of multiple studies, and reporting of standard errors to understand the reliability of that repellent compound to provide the observed protection.

**Table 3:** Guidelines on repellent testing adapted from [78]

WHOPES approved repellent testing methodology
**Laboratory Testing**
Use 20% deet in ethanol as a positive comparison
Human subjects preferable to reflect the end user
Before the test the test area of skin should be washed with unscented soap then rinsed with 70% ethanol / isopropyl alcohol
Mosquitoes should be reared under standard 27 ± 2 C temperature, ≥80 ± 10% relative humidity, and a 12:12 (light:dark) photoperiod.
Mosquitoes should be 3 to 5 days old, nulliparous females, starved for 12 hours preceding the test
Tests should be conducted with three or more species
40 x 40 x 40 cm cages with 50 – 100 mosquitoes for effective dose testing
40 x 40 x 40 cm cages with 200 - 250 mosquitoes for complete protection time testing
Control arms should be used to estimate mosquito readiness to feed
Treatment arms should be offered to mosquitoes after avidity has been measured
**Field Testing**
Use 20% deet in ethanol as a positive comparison
Human subjects preferable to reflect the end user
Before the test the test area of skin should be washed with unscented soap then rinsed with 70% ethanol / isopropyl alcohol
Volunteers should sit >20 metres apart
Design should be completely randomised
Trials should be conducted with medium biting pressures of representative vector species
All participants should be recruited on informed consent from the local area and be provided with malaria prophylaxis
In all testing monitoring of adverse effects should be carried out

## Some fallacies about plant based or natural repellents

It is commonly assumed that plant-based repellents are safer than DEET because they are natural. However, some natural repellents are safer than others, and it cannot be assumed that natural equates to safe [18]. DEET has undergone stringent testing and has a good safety profile. An estimated 15 million people in the U.K., 78 million people in the U.S.A. [82], and 200 million people globally use DEET each year [83]. Provided that DEET is used safely, i.e. it is applied to the skin at the correct dose (such as that in a commercial preparation) and it is not swallowed or rubbed into the mucous membranes then it does not cause adverse effects [84]. DEET has been used since 1946 with a tiny number of reported adverse effects, many of which had a history of excessive or inappropriate use of repellent [85,86]. Its toxicology has been more closely scrutinized than any other repellent, and it has been deemed safe for human use [82,87], including use on children [88], pregnant women [89], and lactating women [84]. In contrast, plant-based repellents do not have this rigorously tested safety record, with most being deemed safe because they have simply been used for a long time [90]. However, many plant-based repellents contain compounds that should be used with caution (Table 1).

It is also commonly stated that plant based repellents are better for the environment than synthetic molecules. While plant volatiles are naturally derived, distillation requires biomass energy, extraction commonly uses organic solvents that must be disposed of carefully, growing the plants uses agrichemicals, such as fertilizers and pesticides (unless sourced from a sustainable and organic source). However, if carefully practiced, cash cropping of plants used for repellents provides a vital source of income for small scale farmers in developing countries [91] and can have beneficial environmental impact when planted in intercropping systems to prevent soil erosions [92]. Therefore, it is important to carefully source of repellent plants to avoid pitfalls associated with unsustainable cropping practices. Another common misconception is that garlic is an effective repellent. It does have a moderate repellent effect when rubbed on the skin [93], although there are far more effective repellents available that also have a more pleasing odour. The consumption of garlic however, has not been shown to be effective at repelling mosquitoes.

## Promising developments in plant based repellents

The field of plant-based repellents is moving forward as consumers demand means of protection from arthropod bites that are safe, pleasant to use and environmentally sustainable. Perhaps the most important consideration is improving the longevity of those repellents that are effective but volatile such as citronella. Several studies looked at improving formulations of plant oils to increase their longevity through development of nanoemulsions [35,94], improved formulations and fixatives [95-97]; while alternate uses such as spatial activity [98-102] and excitorepellency [103,104] have also been investigated. There has been a single clinical study of PMD to lower malaria incidence [26]. This is an exciting discovery since PMD may be recovered from distillation of leaves of *E. citroidora* or chemical modification of citronellal [105]– available from plants of the genus *Cymbopogon.* These plants are already commercially cropped in malaria endemic countries including South America, especially Brazil (6 million trees), southern China, India, Sri Lanka, Congo (Zaire), Kenya and most countries in southern Africa, where it is grown for essential oil production and timber [106]. Local production of insect repellent would remove the high cost of importation in developing countries.

New developments have also been seen in understanding the function of plant-based repellents in insects. Several studies have investigated the behavioural mode of action of repellents through structure-activity studies of contact versus spatial repellency [107], or olfactometry that demonstrated that DEET inhibited mosquito response to human odour whereas *Ocimum forskolei* repels but does not inhibit response to human odour [108]. A further study demonstrates that citronellal directly activates cation channels [10], which is similar to the excitorepellent effect of pyrethrin – another plant based terpine [109], but contrasts with the inhibition effect of DEET [3].

The field of repellent development from plants is extremely fertile due to wealth of insecticidal compounds found in plants as defences against insects [2]. The modern pyrethroids that are the mainstay of the current malaria elimination program that is making excellent progress [110], are synthetic analogues based on the chemical structure of pyrethrins, discovered in the pyrethrum daisy, *Tanacetum cinerariifolium* from the Dalmation region and *Tanacetum coccineum* of Persian origin. The insecticidal component comprising six esters (pyrethrins) is found in tiny oil-containing glands on the surface of the seed case in the flower head to protect the seed from insect attack. Pyrethrins are highly effective insecticides, that are relatively harmless to mammals [111], although it must be emphasised that many other plant produce compounds that are highly toxic to mammals and / or irritating to the skin, and natural does not equate to safe. In the past few years, a plant derived repellent, PMD has been proven to be suitably efficacious and safe to compete with DEET in the field of disease prevention, and repellents have been recognised by WHO as a useful disease prevention tool to complement insecticide-based means of vector control. The field of plant-based repellent evaluation and development had become far more rigorous in recent years and developments in methods of dispensing plant-based volatiles means that extension in the duration of repellency and consequent efficacy of plant-based repellents will be possible in future.

## Author’s contributions

Manuscript drafted by MFM and SJM.

## Competing interests

The authors declare that they have no competing interests
